
JOURNAL INFORMATION
==============================
NLM Title Abbreviation: Wirtschaftsdienst
Journal ID: Wirtschaftsdienst
NLM Journal ID: 101086612

Wirtschaftsdienst (Hamburg, Germany : 1949)

ISSN: 0043-6275

ARTICLE INFORMATION
==============================
PMCID: PMC8800434
PMID: 35125546
DOI: 10.1007/s10273-022-3084-9
Article ID: 3084
Article version: 1
Subjects: Kommentare

Krisenmanagement: Schönwetteransatz

Crisis management: Fair-weather approach

Beretta Edoardo edoardo.beretta@usi.ch

grid.29078.34 0000 0001 2203 2861 Facoltà di Scienze economiche, Università della Svizzera italiana, Via Buffi 13, 6900 Lugano, Schweiz
Electronic publication date: 2022 Jan 29
Print publication date: 2022
Volume: 102
Issue: 1
First page: 9
Last page: 9
Copyright: © Der/die Autor:in 2022
License: Open Access: Dieser Artikel wird unter der Creative Commons Namensnennung 4.0 International Lizenz veröffentlicht (https://creativecommons.org/licenses/by/4.0/deed.de).
Open Access wird durch die ZBW — Leibniz-Informationszentrum Wirtschaft gefördert.
License URL: https://creativecommons.org/licenses/by/4.0/

Keywords: E17, H12, I18
issue-copyright-statement: © The Author(s) 2022

==============================

Wirtschaftsdienst, 2022, 102(1), 9
JEL: E17, H12, I18

Krisenmanagement

### Schönwetteransatz

Die Prognose, nach der die SARS-CoV-2-Pandemie nach globalem Wüten in eine Endemie umschlagen könnte, macht seit der Omikron-Variante die Runde und wird als Hoffnungsschimmer in Sachen schwerer Krankheitsverläufe betrachtet. Doch egal, wie man sie schönreden mag: Sie ist auch Zeugnis menschlichen Versagens. Falls COVID-19 sich bald wie eine saisonale Erkrankung à la Grippe (von der allerdings schon seit Hippokrates Aufzeichnungen aus dem 5. Jahrhundert v. Chr. die Rede ist) verhalten würde, wäre es trotzdem kein Grund zum Feiern. Während die Pharmaindustrie das Unmögliche in Rekordzeit möglich gemacht hat, haben sich weite Teile von Politik (inklusive zuständiger internationaler Behörden) und Gesellschaft allzu oft fehlerhaften Denkmustern hingegeben: Die Politik hat einerseits kommunikativ schwer zu vermittelnde Regeln formuliert und die Impfpflicht, nämlich die strukturell einzige Lösung gegen die Pandemie, a priori ausgeschlossen (um sie jetzt notgedrungen wieder aufs Tapet zu bringen), und die Gesellschaft hat ohne jegliche wissenschaftliche Kenntnisse oft einfachste Schutzmaßnahmen wie das Tragen einer Gesichtsmaske infrage gestellt. Dass man immer noch darüber diskutieren muss, wie oder ob sich Impfverweigernde überzeugen ließen, spricht Bände über die bedingte menschliche Rationalität. Denn dieser ist der gemeinsame Nenner vieler Fehleinschätzungen: Die trügerische Einstellung (ein Denkfehler also), dass das Wetter sozusagen immer schön sein und nie umschlagen würde. Doch kommt es nun mal zu unerwarteten Situationen – umso mehr in einer globalen Gesellschaft wegen deren beschleunigten Treibens –, die sich nur bei ausreichender Krisenvorsorge bewältigen lassen. Viel zu oft verfällt man aber dem Reiz des „Schönwetterdenkens", um erst spät zu erkennen, dass für unmöglich Gehaltenes doch nicht so unwahrscheinlich ist.

So ist es mit der noch Anfang 2020 von allen öffentlich zugänglichen Risikoberichten unvorhergesehenen Corona-Pandemie (deren Ursprung noch gründlicherer wissenschaftlicher Recherche bedarf) geschehen, als Regierungen nach Überschwappen der ersten Ansteckungswelle nach Europa feststellen mussten, dass die schnelle Güterversorgung (etwa mit Arztkitteln, Mundschutzmasken für die breite Bevölkerung usw.) im Falle von Lockdowns oder vorsorglicher Hortung in den Herstellerregionen wie China fast zum Erliegen kommen könnte. Wenn bei „schönem Wetter" Ökonom:innen gebetsmühlenartig vor zu hohen Lagerkosten gewarnt und auf die Alternative der internationalen Beschaffung benötigten Equipments hingewiesen haben, sind bei „schlechtem Wetter" möglichst lokal aufzustockende Lagerbestände essenziell. Die Corona-Seuche hat also wieder vor Augen geführt, dass man sowohl mikro- (d. h. im Einzelbereich) als auch makroökonomisch (d. h. im Gesamtbereich) gewappnet zu sein hat: Eigene Autos bleiben beispielsweise trotz Nachhaltigkeitsdebatte wichtig, um bei steigenden Fallzahlen öffentliche Transportmittel zu meiden. Auch kleine bzw. nicht weit zu öffnende Fenster in öffentlich zugänglichen Gebäuden mögen wärmedämmend sein, verhindern aber Kreuz- und Stoßlüften. Man hat eben nicht genügend um die Ecke (oder *out of the box*) gedacht.

Die globale Finanzkrise ab 2007, die hinsichtlich des sinkenden globalen BIP (-1,3 % im Jahr 2009) jüngst nur vom coronabedingten Wirtschaftseinbruch um -3,4 % im Jahr 2020 übertroffen wurde, lässt sich aber auch auf den (allerdings reizenden) Irrglauben zurückführen, dass Kreditnehmende ohne ausreichende Bonität Darlehen zurückzahlen, Banken sowohl in guten als auch schlechten Konjunkturphasen von übermäßiger Geldschöpfung profitieren sowie Wirtschaftssubjekte im Allgemeinen (da als Homines oeconomici zu verstehen) es irgendwie immer richten würden. Davon kann allerdings nicht a priori ausgegangen werden und – falls unbedingt doch – ist die Bewältigung jedes nicht eingeplanten Krisenszenarios mit Opfern und Kosten im weitesten Sinne verbunden. Es gibt etliche Beispiele, wie oft man dem „Schönwetteransatz" bei wirtschaftspolitischen Entscheidungen verfällt: Bemühungen zur Staatsschuldensenkung sind lange zu kurz ausgefallen – in der EU und Eurozone stagnierten sie noch 2019 (trotz guter Konjunktur) bei jeweils 77,2 % und 83,6 %, um schon 2020 bis jeweils 90,1 % und 97,3 % gegenüber dem BIP zu steigen – oder auch Inflation aufgrund der Aufholjagd globaler Nachfrage, aber auch des Revanchier-Eifers hinsichtlich der Umsätze der Unternehmen nach den Lockdowns haben bis vor Kurzem nur die Wenigsten kommen sehen. Und das ungeachtet allerlei typischer Teuerungstrends nach Naturkatastrophen, Waldbränden usw. Selbst Individuen haben sich vor Notsituationen zu schützen: Sie tun es aber beispielsweise kaum, solange der globale Haushaltsverschuldungstrend anhält.

Die Wirtschaftsgeschichte – egal, ob nah oder weit zurückliegend – lehrt, dass Krisenvorbeugung besser als -management ist, aber auch dass beide Mangelware sind.

Edoardo Beretta
Università della Svizzera italiana

© Der/die Autor:in 2022. Open Access: Dieser Artikel wird unter der Creative Commons Namensnennung 4.0 International Lizenz veröffentlicht (creativecommons.org/licenses/by/4.0/deed.de).

Open Access wird durch die ZBW – Leibniz-Informationszentrum Wirtschaft gefördert.
